# Decrease of choriocapillary vascular density measured by optical coherence tomography angiography in Vogt-Koyanagi-Harada disease

**DOI:** 10.1007/s00417-021-05238-5

**Published:** 2021-07-03

**Authors:** Anyi Liang, Shanshan Jia, Fei Gao, Xiaoxu Han, Minghang Pei, Yi Qu, Junyan Xiao, Chan Zhao, Meifen Zhang

**Affiliations:** 1 Department of Ophthalmology, Peking Union Medical College Hospital (Dongdan Campus), Chinese Academy of Medical Sciences, No.1 Shuaifuyuan Wangfujing Dongcheng District, Beijing, 100730 China; 2 Department of Ophthalmology, The Second Clinical Medical College (Shenzhen People’s Hospital), Jinan University, Shenzhen, 518020 China; 3 Department of Ophthalmology, First Affiliated Hospital of Zhengzhou University, Zhengzhou, 450052 Henan China; 4 Department of Ophthalmology, Peking University Third Hospital, Beijing, 100191 China; 5 Key Laboratory of Ocular Fundus Diseases, Chinese Academy of Medical Sciences, Beijing, China

**Keywords:** Vogt-Koyanagi-Harada disease, Optical coherence tomography angiography, Choriocapillary vascular density, Disease stage, Visual acuity

## Abstract

**Purpose:**

Changes of choroidal circulation throughout the disease course of Vogt-Koyanagi-Harada (VKH) disease and the clinical significance remain unclear. Choriocapillary vascular density (CC VD) measured by optical coherence tomography angiography (OCTA) were compared in different disease stages of VKH and its correlation with other parameters was analyzed, aiming to explore their clinical relevance.

**Methods:**

This is a retrospective case series. One hundred and fourteen VKH patients and 47 normal controls (NCs) were included. Patients were grouped into the acute uveitic, convalescent, and chronic recurrent stages (only anterior recurrent cases included), and OCTA images were obtained from VKH patients in these stages. Best corrected visual acuity (BCVA), CC VD, and subfoveal choroidal thickness (SFCT) were recorded and compared.

**Results:**

CC VD in acute (58.26% ± 0.84%), convalescent (64.85% ± 0.33%), and chronic recurrent (62.78% ± 0.70%) stage of VKH patients were all significantly lower than that in NCs (66.37% ± 0.41%) (*p* < 0.001, *p* = 0.017, and *p* < 0.001, respectively). CC VD increased by 6.59% ± 0.91% with resolution of acute inflammation (*p* < 0.001) and decreased by 2.07% ± 0.74% during anterior uveitis relapse (*p* = 0.009). Patients with a positive history of anterior recurrence had lower CC VD (− 2.43% ± 0.75%, *p* = 0.003) in the convalescent stage than those without. CC VD was negatively correlated with logMAR BCVA in VKH (*r* =  − 0.261, *p* < 0.001).

**Conclusion:**

CC VD was decreased in every stage of VKH. CC VD has the potential to reflect the status of uveitis and might be promising in monitoring the disease activity. OCTA is a convenient and straightforward tool to evaluate choroidal vascularity, and CC VD provides supplemental quantitative information of the choriocapillaris. Further studies are needed to explore the values of OCTA quantitative parameters in monitoring VKH progression, predicting visual prognosis, and guiding clinical decisions.


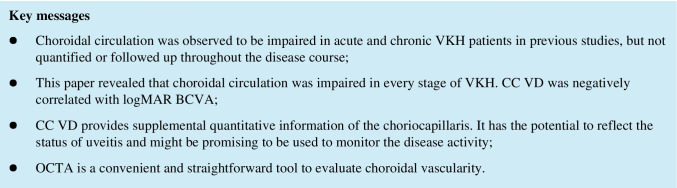


## Introduction

Vogt-Koyanagi-Harada (VKH) disease is a T cell-mediated multisystemic, granulomatous autoimmune disorder with ocular involvement in the form of bilateral granulomatous panuveitis [1]. Repeated episodes of uveitis may cause severe complications and poor visual outcome [2–7]. Notably, with the help of imaging modalities including enhanced depth imaging optical coherence tomography (EDI-OCT) [8, 9] and indocyanine green angiography (ICGA) [10], choroidal changes of VKH can be viewed and partially quantified.

Advances in optical coherence tomography angiography (OCTA) have allowed in vivo visualization and quantitative measurement of retinal and choroidal vasculatures with high repeatability and reproducibility. In the area of uveitis, OCTA is becoming increasingly important not only as a diagnostic imaging modality supplementing traditional techniques, but also as a means to provide quantitative evaluations during follow-up [11]. Choriocapillary (CC) blood flow and vascular densities (VD) were found to be significantly decreased in both acute and chronic VKH patients in several recent studies of ours and other teams [12–14], indicating substantial choroidal circulation impairment. But how the choroidal blood flow changes throughout the disease course and whether there is any clinical significance remain unclear, and there have been very few studies on these topics.

To address this, we used OCTA to measure and compare CC VD in different disease stages of VKH and analyzed its correlation with other parameters, aiming to explore their clinical relevance.

## Materials and methods

### Study participants

This is a retrospective case series. Our study was approved by the Institutional Review Board of Peking Union Medical College Hospital (PUMCH) and followed the tenets of Declaration of Helsinki. Informed consents were obtained from all participants for using their clinical data. OCTA images and medical records of VKH patients who underwent OCTA scans in PUMCH from October 2015 to December 2019 and normal controls (NC) were analyzed. Eyes with refractive errors greater than 6.00 diopters and other confounding chorioretinal pathologies were excluded. The diagnosis of VKH disease was based on the American Uveitis Society’s revised international criteria [15], which was applied to each patient. Participants were grouped into the acute uveitic stage, convalescent or chronic stage, and chronic recurrent stage according to the classic Moorthy criteria [16].

### Measurements of vascular densities by OCTA

**Fig. 1:**
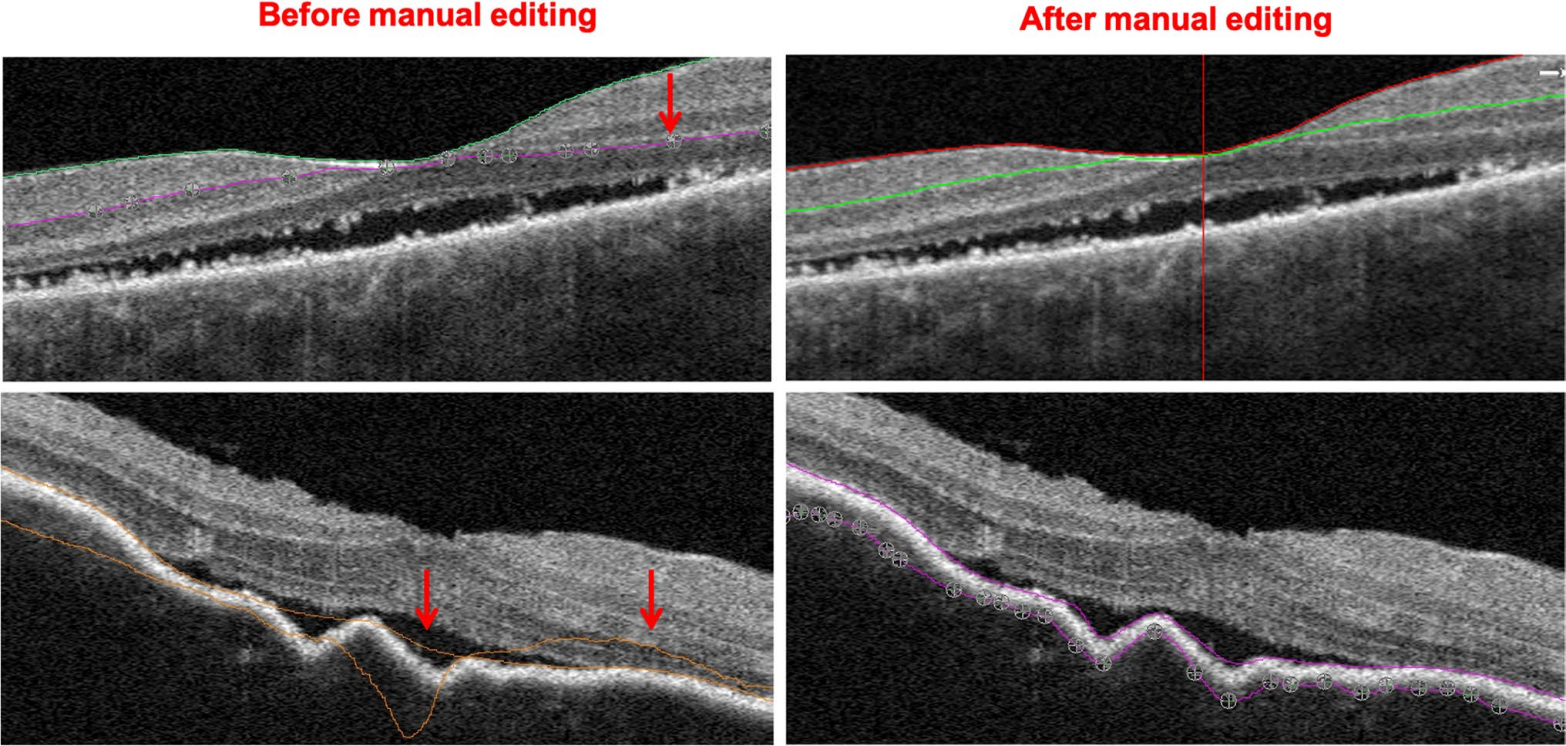
Pictures demonstrating the segmentation before and after manual correction. Red arrows: locations of erroneous segmentation

OCTA images were obtained with AngioVue (Optovue, Fremont, CA, USA). Segmentation and quantification of the vascular plexus were performed automatically by the built-in software. Serous retinal detachment and choroidal folds are common in the acute stage, which cause difficulties and compromise accuracy in the measurement of CC VD by introducing errors in segmentation of the choriocapillaris. So, every OCTA image was proofread and manually edited with the built-in “Segmentation editing and automatic propagation” function of the device to improve accuracy when necessary (Fig. 1). The built-in “follow-up” program was used to ensure that the same OCTA sections were obtained in repeated examinations. Qualities of the images were graded automatically by the device as 10 levels, from Q1 (1/10, the worst) to Q10 (10/10, the best), and only scans with qualities of Q6 or above were included in this study.

**Fig. 2:**
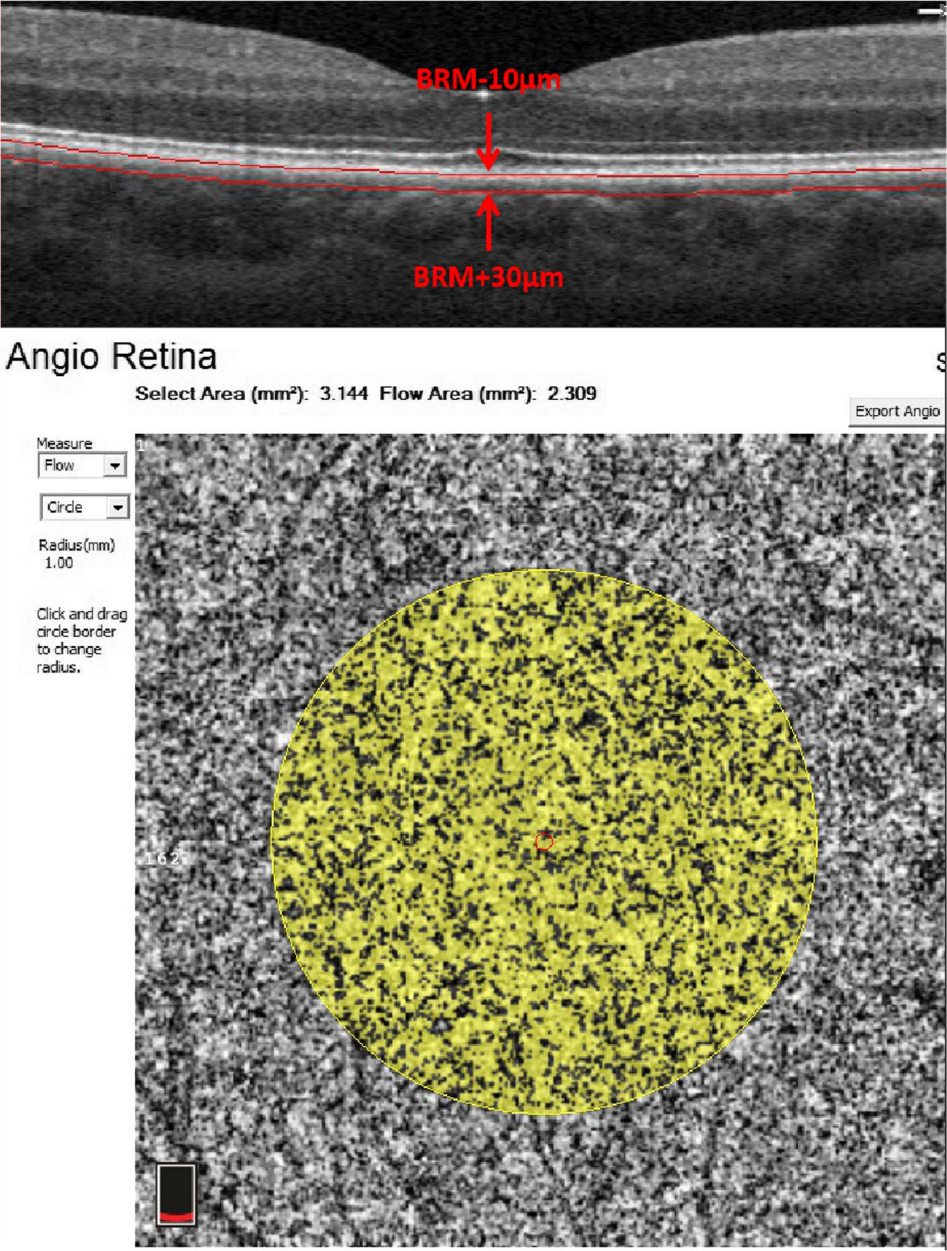
Illustration of choriocapillary vascular density measurement. Top: segmentation standard. Area between 2 red lines stands for choriocapillaris. Bottom: vascular density measurement. The total area of the yellow circle is the selected area (SA, mm^2^); signals highlighted in yellow within the circle stands for blood flow area (FA, mm^2^); black within the circle indicates area without blood flow signal. CC VD is calculated as FA/SA (%). BRM: Bruch’s membrane

**Fig. 3:**
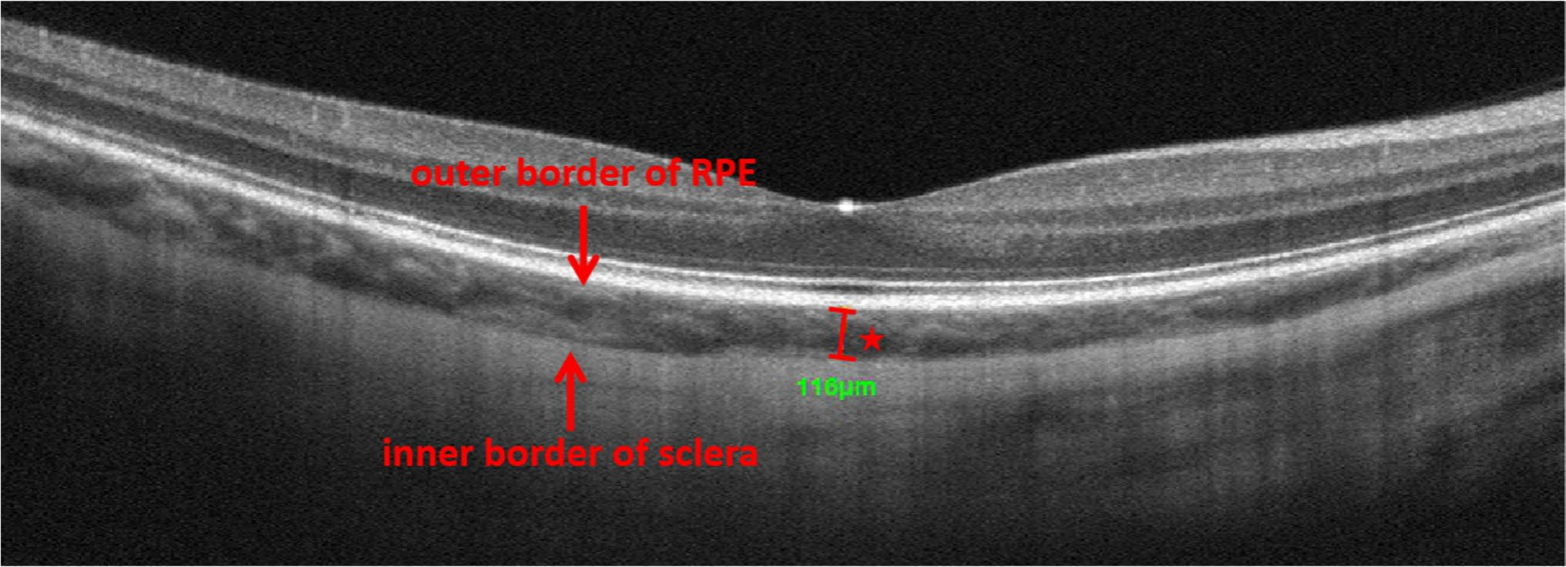
Illustration of subfoveal choroidal thickness measurement. SFCT is measured as the vertical distance indicated by the red star. RPE: retinal pigment epithelium

Scans from eyes with active (either at the acute uveitic or the anterior uveitis recurrent stage) or inactive (at the convalescent stage) inflammation were selected for analysis. Best corrected visual acuity (BCVA) in logMAR units (recorded as logMAR BCVA), choriocapillary vascular density (CC VD), and subfoveal choroidal thickness (SFCT) were recorded for all participants. CC VD was measured in the “macular, 3*3 mm” scanning mode. CC was defined as from 10 μm above Bruch’s membrane (BRM) to 30 μm below BRM and CC VD was calculated as the percentage of flow area (FA) over selected area (SA) (illustrated in Fig. 2). SFCT was measured under the fovea from the outer border of the retinal pigment epithelium (RPE) to the inner border of sclera (illustrated in Fig. 3). For choroidal thickness that was beyond measurement limit of the OCTA system, 800 μm was recorded as the value according to the strategies of previous studies [8, 17, 18].

### Statistical analysis

IBM SPSS Statistics (Armonk, NY, IBM Corp.) version 22.0 and GraphPad Prism® (GraphPad Software Inc., La Jolla, CA) version 6.01 were used for statistical analysis and graphs presentation, respectively. A *p* value equal to or less than 0.05 was considered significant for all statistical testing. CC VD and SFCT changes during the disease course and between patients and controls were performed using nonparametric test. The correlations between VD and other parameters were analyzed with Pearson correlation coefficient.

## Results

### Study participants

A total of 114 VKH patients (52 males, 62 females, 217 eyes) and 47 age-matched NCs (24 males, 23 females, 94 eyes) were enrolled in the study. Eleven eyes from 11 patients were excluded because the quality of their OCTA images was poor. The mean ages of VKH patients and NCs were 41.0 ± 1.3 years (9–69 years) and 41.2 ± 1.8 years (14–64 years), respectively (*p* = 0.956). Ninety-four OCTA scans from NCs and 726 from VKH patients were reviewed and analyzed, among which 135 (18.6%) were obtained in the acute stage, 540 (74.4%) in the convalescent stage, and 51 (7.0%) in the chronic anterior recurrent stage. For patients who had more than 1 OCTA scans in the same stage, quantitative measurements of images from different time points in that stage were averaged.

### Choriocapillary vascular densities and choroidal thickness in VKH and NCs

**Fig. 4:**
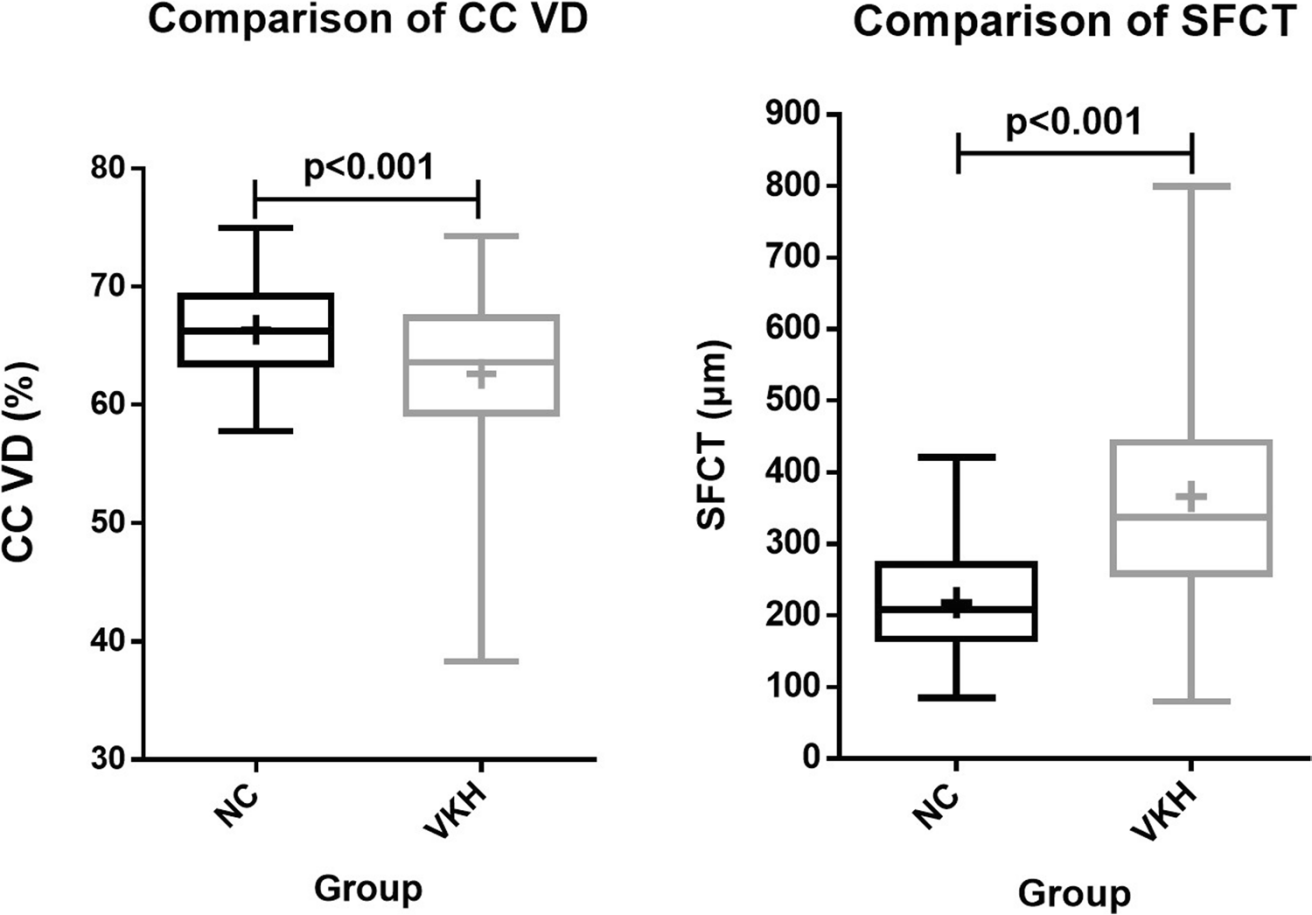
Comparisons of CC VD and SFCT between VKH and NCs. CC VD decreased and SFCT increased in VKH patients compared to NCs

CC VD was 62.64% ± 0.36% in VKH patients (regardless of stages), which was significantly lower than that in NCs (66.37% ± 0.41%, *p* < 0.001). SFCT was 366.69 μm ± 9.15 μm in VKH patients (regardless of stages), which was significantly thicker than that in NCs (218.30 μm ± 6.91 μm, *p* < 0.001) (Fig. 4).

**Fig. 5:**
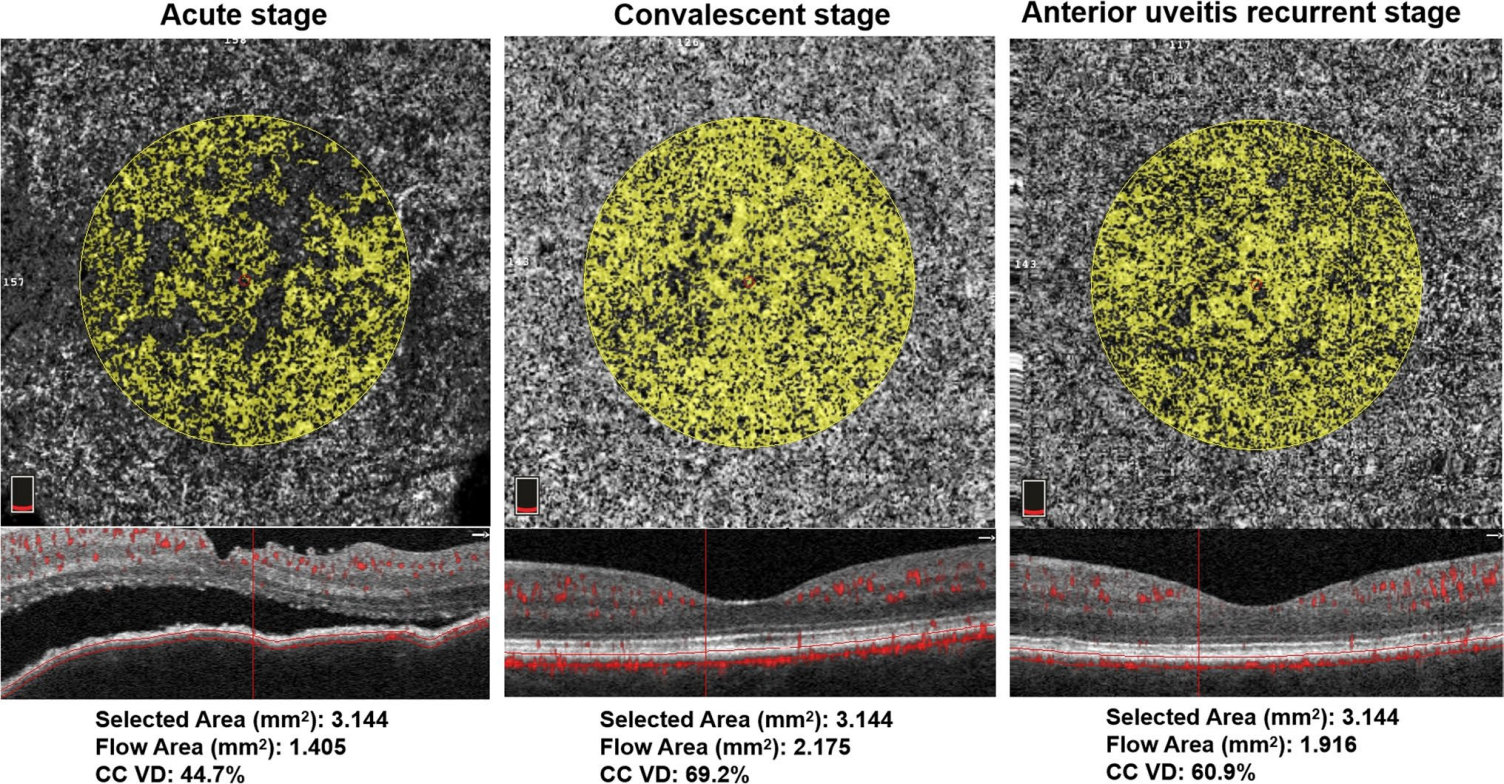
Demonstration of representative images of CC VD measurement (from the same eye of the same patient)

Representative images of choriocapillary layer with CC VD measurements in different stages of VKH are shown in Fig. 5.

CC VD, SFCT, and LogMAR BCVA in different stages of VKH patients and in NCs are shown in Table 1. Compared to NCs, CC VD was significantly decreased in the acute stage (− 8.11% ± 0.94%; 95% CI: − 6.25%, − 9.97%; *p* < 0.001), convalescent stage (− 1.52% ± 0.54%; 95% CI: − 2.59%, − 0.45%; *p* = 0.017), and chronic anterior recurrent stage (− 3.59% ± 0.77%; 95% CI: − 5.10%, − 2.07%; *p* < 0.001); SFCT was significantly increased in the acute stage (increased by 278.81 μm ± 20.33 μm; 95% CI: 238.54 μm, 319.09 μm; *p* < 0.001), convalescent stage (increased by 81.51 μm ± 10.83 μm; 95% CI: 60.19 μm, 102.83 μm; *p* < 0.001), and chronic anterior recurrent stage (increased by 146.19 μm ± 19.33 μm; 95% CI: 107.48 μm, 184.90 μm; *p* < 0.001) compared to the NC.

**Table 1:** CC VD, SFCT, and LogMAR BCVA in VKH and NCs

Parameters	Acute uveitic stage (*n* = 90)	Convalescent stage (*n* = 175)	Chronic anterior recurrent stage (*n* = 46)	Normal control (*n* = 94)
CC VD (%)	58.26 ± 0.84 (38.3, 72.8)	64.85 ± 0.33 (54.7, 74.3)	62.78 ± 0.70 (53.4, 72.7)	66.37 ± 0.41 (57.8, 75.0)
SFCT (μm)	497.11 ± 19.12 (163, 800)	299.80 ± 8.33 (80, 690)	364.49 ± 18.06 (162, 642)	218.30 ± 6.91 (85,421)
logMAR BCVA	0.32 ± 0.03 (− 0.13, 1.10)	0. 11 ± 0.02 (− 0.10,1.15)	0.19 ± 0.04 (0.00, 1.00)	NA

All the values were presented as mean ± standard error (minimum, maximum)

### Changes of vascular densities, choroidal thickness, and visual acuity over different VKH stages

**Fig. 6:**
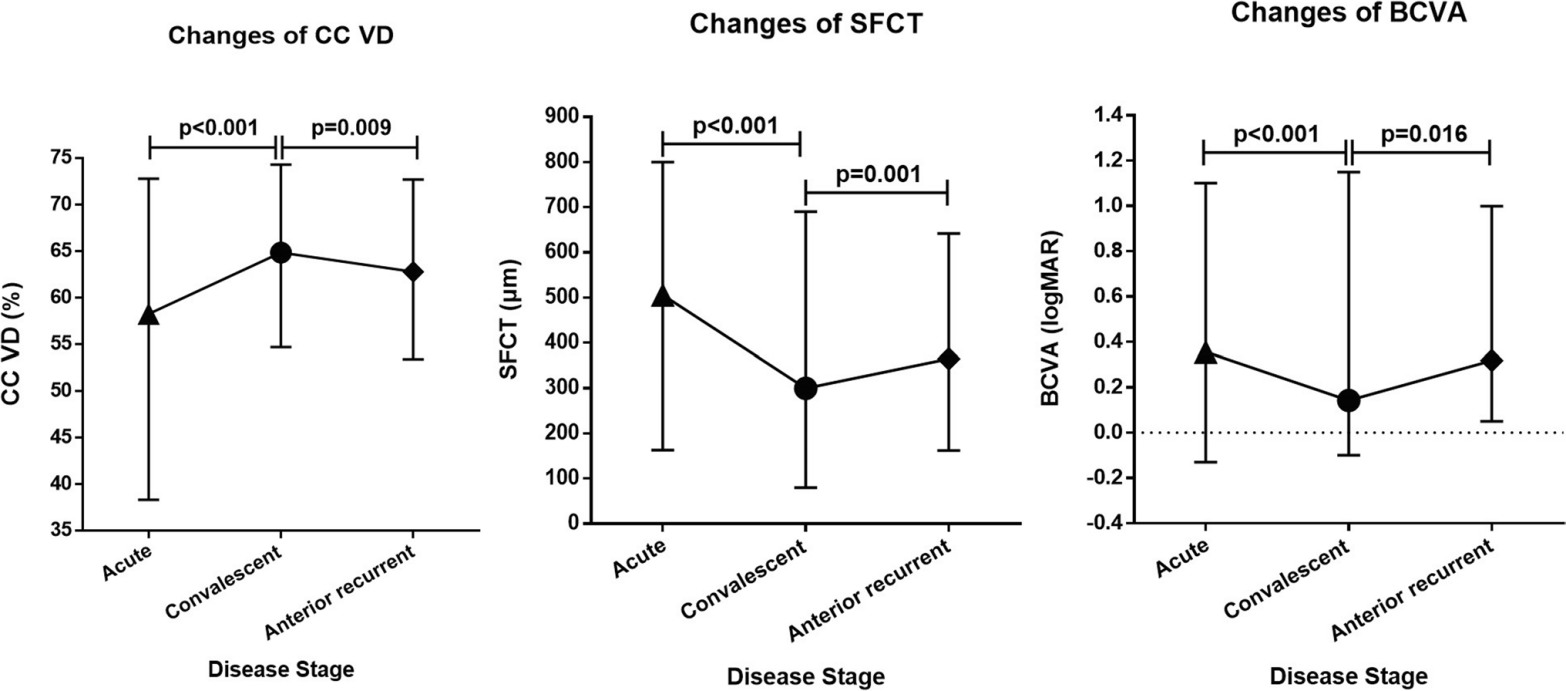
Dynamic changes of CC VD, SFCT, and LogMAR BCVA over different disease stages of VKH. CC VD increased with uveitis resolution and decreased during anterior uveitis recurrence. SFCT decreased with uveitis resolution and increased during anterior uveitis recurrence. LogMAR BCVA decreased with uveitis resolution and increased during anterior uveitis recurrence

Figure 6 showed the dynamic changes of CC VD, SFCT, and LogMAR BCVA over different VKH stages. With resolution of acute inflammation, CC VD increased by 6.59% ± 0.91% (95% CI: 4.08%, 8.39%; *p* < 0.001); SFCT decreased by 197.31 μm ± 20.85 μm (95% CI: 156.03 μm, 238.58 μm; *p* < 0.001); LogMAR BCVA decreased by 0.21 ± 0.04 (95% CI: 0.14, 0.29; *p* < 0.001). During anterior uveitis relapse, CC VD decreased by 2.07% ± 0.74% (95% CI: 0.62%, 3.52%; *p* = 0.009); SFCT increased by 64.68 μm ± 18.78 μm (95% CI: 27.67 μm, 101.70 μm; *p* = 0.001); logMAR BCVA increased by 0.08 ± 0.04 (95% CI: 0.00, 0.16; *p* = 0.016).

### Comparison between patients with and without histories of anterior uveitis recurrence

Among the 114 VKH patients included in this study, 63 had been followed up for more than 1 year and were further grouped into with or without a history of anterior uveitis recurrence. OCTA scans of these patients obtained in the convalescent stage were analyzed. Results showed that compared to patients who never had anterior recurrences during the disease course (*n* = 30), those who had a positive history of anterior recurrence (*n* = 33) had lower CC VD (− 2.43% ± 0.75%, *p* = 0.003), while the differences of SFCT (− 1.03 μm ± 20.23 μm, *p* = 0.97) and logMAR BCVA (0.05 ± 0.03, *p* = 0.102) were not statistically significant (Table 2).

**Table 2:** Comparison between patients with and without histories of anterior uveitis recurrence

History of anterior uveitis recurrence	Number of patients	CC VD (%)	SFCT (μm)	LogMAR BCVA
Positive	33	63.72 ± 4.35 (54.8, 71.8)	309.92 ± 109.98 (124, 571)	0.08 ± 0.19 (− 0.09, 0.98)
Negative	30	66.06 ± 3.65 (54.8, 72.4)	310.95 ± 108.85 (85, 690)	0.03 ± 0.13 (− 0.10, 0.70)
Difference	NA	− 2.43 ± 0.75 (− 3.82, − 0.87)	− 1.03 ± 20.23 (− 41.11, 39.04)	0.05 ± 0.03 (− 0.02, 0.11)
*p* value	NA	**0.003**	0.97	0.102

All the values were presented as mean ± standard error (minimum, maximum)

The bold data mean that the *p* values are significant (<0.05)

### Correlation between vascular density, choroidal thickness, and visual acuity

Correlation analysis revealed that CC VD was negatively correlated with logMAR BCVA in VKH. When analyzed in different stages separately, CC VD was negatively correlated with logMAR BCVA only in the acute uveitic stage, but not in other stages. SFCT was not significantly correlated with logMAR BCVA in any stage. CC VD appeared to be negatively correlated with SFCT (Table 3).

**Table 3:** Correlation analyses between CC VD, SFCT, and logMAR BCVA in VKH patients

Stage		3 stages		Acute		Convalescent		Anterior recurrent	
		CC VD	SFCT	CC VD	SFCT	CC VD	SFCT	CC VD	SFCT
BCVA	***r***	− **0.261**	0.096	− **0.244**	− 0.19	− 0.046	− 0.093	0.059	− 0.068
	***p***	** < 0.001**	0.124	**0.036**	0.103	0.594	0.276	0.708	0.67
CC VD	***r***		− **0.419**		− **0.326**		0.007		− 0.142
	***p***		** < 0.001**		**0.002**		0.923		0.352

The bold data mean that the *p* values are significant (<0.05)

## Discussion

To the best of our knowledge, there have been few researches on the quantitative analysis of choroidal circulation in VKH so far. This is the first study using OCTA to investigate choroidal blood flow changes in different stages of VKH. Furthermore, we are pioneering in analyzing the correlation between vascular density and thickness of the choroid and their correlation with vision. Besides, the sample size in this study was relatively large.

Choroid is the main site of ocular inflammation in VKH disease [1], so characterization of the pathophysiological features of the choroid would be of great significance to the diagnosis, follow-up, and evaluation of treatment effectiveness. Morphological changes are mostly straightforward and apparent, but quantification analysis would be more accurate in reflecting the severity and changes of inflammation. Choroidal thickness (CT) and blood circulation are the 2 main quantification indexes. Most of the reports about CT are consistent with our study, where we detected a dramatically thickened choroid in the acute uveitic, convalescent, and anterior uveitic recurrent stages of VKH. The CT was reported to increase in the acute phase [19], subside with effective treatment [17], and rebound with uveitis recurrence [18] in previous studies, the same changing mode as that in our study. These findings indicated that thickening of the choroid is one of the hallmarks of VKH. CT can be used as a quantifiable marker of disease severity as it changes sensitively with the aggravation or resolution of inflammation.

ICGA is a classic and valuable tool to evaluate vessel conditions in the choroid, which can reflect choroidal hypoperfusion and vascular leakages in VKH [20]. It can be used to semi-quantify and evaluate the choroidal blood flow by calculating the number and total area of hypofluorescent dark dots (HDDs) [10]. Combined with FFA, ICGA can also quantify the severity of uveitis by scoring the signs of uveitis [21]. However, because of its invasiveness, dye-related complications, and inability to directly quantify vascular densities accurately, other techniques were used to supplement the functions of ICGA. Decrease of choroidal vascularity index (CVI) and blood flow velocity in active VKH were detected with EDI-OCT and laser speckle flowgraphy (LSFG), respectively [10], indicating circulation impairment of the choroid. In this study, we adopted the latest blood flow quantification method, OCTA, to evaluate the circulation, which is more convenient and straightforward. OCTA cannot detect the signals of flow velocity below a certain limit [22], i.e., too slow or static, so what was measured might reflect the actual effective circulatory amount, which makes the measurement more meaningful. In contrast, in Liu et al.’s study [9], CVI was higher in VKH than that in NC even though choroidal circulation was impaired, because the increased blood in choroid might be static.

CC VD of VKH was found to be lower than that of NC in every disease stage, indicating choroidal circulation abnormality throughout the whole disease course. The impairment of choroidal blood flow was confirmed in various studies with different techniques, including hypofluorescent dark dot and decreased dye filling velocity of the choroid on ICGA [10, 23], decreased choroidal blood flow velocity on LSFG, decreased CVI on EDI-OCT [9], and lower CC VD on OCTA [12]. Notably, we noticed that even in the chronic convalescent stage when inflammation was inactive, CC VD was still lower than that of NC. One of the reasons might be that damages to the choroidal blood flow by inflammation were persistent and partially irreversible. Another possible explanation is that even the uveitis appeared to be inactive clinically, potential subclinical inflammatory activity might exist and continue to damage the choroidal circulation. Nongranulomatous inflammatory cell infiltration and atrophy of the choriocapillaris during convalescent stage in pathological observation might also explain the circulation defect [24].

In our study, CC VD was found to significantly decrease in the acute uveitis stage, increase as the inflammation subsided, and drop again when uveitis relapsed, meaning that choroidal circulation changed in accordance with the inflammation. It has long been reported and accepted that SFCT also change sensitively with the inflammatory activity in VKH eyes. A number of researches reported that SFCT increased in active inflammation and recovered after treatment [8, 17]. Tagawa Y et al. [8] also observed choroid thickening 1 month prior to development of anterior segment inflammation. Correlation analysis revealed that CC VD was negatively correlated with SFCT, meaning that choroidal circulation was worse in thicker choroid in VKH. This might be explained by the pathological changes of the choroid in VKH [24–26]. Large numbers of inflammatory cells infiltrating the choroidal stroma led to stromal edema, which compressed the choriocapillaris and caused blood circulation impairment. The consistency of changing modes of these 2 indexes and their correlation indicated that CC VD might be a novel quantitative marker to evaluate VKH patients.

We discovered that in the anterior uveitis recurrent stage, even if the relapse was in the anterior segment and no inflammatory signs were detected in the fundus, the CC VD was still found to be decreased. It indicated that there might be potential inflammation of posterior segment in anterior uveitis recurrence. Multiple lineages of evidences have revealed that the posterior segment was concomitantly inflamed during anterior uveitis recurrences in VKH patients. Chee SP and colleagues [27] reported that in a series of VKH patients with clinically isolated anterior uveitis recurrence, ICGA revealed early large choroidal vessel leakage and late diffuse leakage, which subsided after intensive immunosuppressive treatment. Using ICGA and LSFG, Kenichi Namba and colleagues [10] later demonstrated subclinical choroidal circulation impairment due to granulomatous inflammation as well as post-treatment improvement in isolated anterior uveitis recurrence in VKH. And this was also evidenced by our finding that SFCT increased in the anterior uveitis recurrent stage.

Although anterior uveitis recurrence is common in the chronic stage of VKH, not every patient would experience recurrent uveitis attacks. In order to find out whether there are any differences between patients with and without anterior recurrence in the chronic convalescent stage and whether these different presentations predict relapse or not, we compared CC VD, SFCT, and BCVA of these 2 groups of patients. Our results showed that patients with a positive history of anterior uveitis recurrence appeared to have lower CC VD than those without. Our hypothesis of this finding is that patients with a history of anterior recurrence had more severe ocular inflammation in the acute phase or less well-controlled disease in the chronic phase, which had resulted in more prominent and prolonged damages to the choroidal vasculature. However, further studies are needed to investigate whether anterior uveitis recurs as a result of CC VD impairment or vice versa.

BCVA is one of the main indicators of visual function. Quantitative OCTA measurements have been reported to be associated with visual outcome in a variety of diseases including diabetic retinopathy, retinal vein occlusion, and glaucoma [15, 28–31]. Similar findings were also reported in Behcet’s uveitis by Cheng D et al. [32] that decreased deep retinal capillary plexus (DCP) VD was found to be correlated with reduced BCVA and disruption of outer retina substructures. So, it is worth exploring whether quantitative measurements by OCTA were correlated with VA or not in VKH. Our study revealed significant negative correlation between CC VD and logMAR BCVA, indicating that choroid quantitative parameters like VD might have the potential to reflect visual function. This result was not beyond expectation because CC VD reflects the perfusion condition of the choroid, which provides blood and metabolic supply for the outer retina. On the other hand, choroidal thickness did not appear to be correlated to visual acuity, indicating that CT was not an independent predictor of VA. Noticeably, visual function was influenced by a number of factors in the diseased state, including refractive media opacity, macular edema, and exudative retinal detachment, so further explorations are needed to find out the influencing factors of visual function in VKH.

The following limitations should be kept in mind to interpret results of the current study: (1) the study was retrospective; thus, biases may exist. (2) Only the CC VD within 3 mm around the fovea was measured, which cannot represent the vasculature more peripherally. (3) The inherent motion and projection artifacts of OCTA may impair the accuracy of data.

## Conclusion

In conclusion, this study confirmed that choroidal circulation was impaired in every stage of VKH. CC VD has the potential to reflect the status of uveitis and might be promising to be used to monitor the disease activity. OCTA is a convenient and straightforward tool to evaluate choroidal vascularity, and CC VD provides supplemental quantitative information of the choriocapillaris. Further studies are needed to explore the values of OCTA quantitative parameters in monitoring VKH progression, predicting visual prognosis, and guiding clinical decisions.
